# The EXPERIENCE system for the investigation of behavioral differences between depressed and healthy-control participants in Virtual Reality

**DOI:** 10.1192/j.eurpsy.2023.986

**Published:** 2023-07-19

**Authors:** S. Sutori, I. Todorov, G. Hadlaczky, V. Carli

**Affiliations:** National Centre for Suicide Research and Prevention (NASP), Karolinska Institutet, Solna, Sweden

## Abstract

**Introduction:**

The EXPERIENCE project aims to enable the creation and sharing of extended-personal realities in virtual reality (VR). Currently, software and hardware technology are under development, that will automatically generate VR environments based on neurophysiological, psychological, cognitive, and behavioral data to support not only the recording of personal experiences but the transmission as well to another user. Potential use cases include enhanced treatment and the assessment of symptom severity of affective disorders.

**Objectives:**

The objective is to design and create a virtual reality environment that enables the identification of between-group differences in behavioral measures when comparing depressed and healthy-control participants.

**Methods:**

We conducted a literature review to identify measures that can be implemented in VR and have the potential to show differences between depressed and healthy-control participants. PubMed and ResearchGate databases were screened to identify potential cognitive tasks. A selection protocol was developed considering effect size, homogeneity of results, risk for cybersickness, cognitive demand, domain heterogeneity, and VR compatibility to choose 4 out of the 47 initial tasks. In addition to the cognitive tasks, behavior measures were considered as well and a virtual environment has been equipped to assess (1) exploratory behavior; (2) engagement with emotionally valenced stimuli (via eye-tracking); (3) metacognitive sensitivity, (4) persistence/grit, and (5) possible effects of mood induction.

**Results:**

Based on the above review, a virtual environment has been developed which is composed of four rooms and a hallway where the starting point is. After an initial tutorial on how the environment/controllers work participants are free to explore and instructions are only provided for the specific cognitive tasks which have to be solved to open the doors and move between rooms. The rooms are equipped with numerous interactive objects and images with varying emotional valence. The engagement with the environment and general activity are continuously recorded and can be retrieved for analyses after participants exit the environment.

**Conclusions:**

If the controlled VR environment will be proven effective for the assessment of depressive symptoms in future studies, the EXPERIENCE system could incorporate direct and objective behavioral measures into the assessment depressive symptoms. Consequently, the system has the potential to support the clinical diagnosis of affective disorders.

**Acknowledgement:**

The EXPERIENCE project is funded by the European Commission H2020 Framework Program with the Grant No. 101017727.

**Disclosure of Interest:**

None Declared

